# Design and optimization of a compact laser-driven proton beamline

**DOI:** 10.1038/s41598-018-24391-2

**Published:** 2018-04-19

**Authors:** M. Scisciò, M. Migliorati, L. Palumbo, P. Antici

**Affiliations:** 1 University of Rome “La Sapienza” & INFN, P.zzale Aldo Moro 5, 00161 Rome, Italy; 2 INRS-EMT, 1650 Boul. Lionel Boulet, J3X 1S2 Varennes, Canada

## Abstract

Laser-accelerated protons, generated by irradiating a solid target with a short, energetic laser pulse at high intensity (*I* > 10^18^ *W*·*cm*^−2^), represent a complementary if not outperforming source compared to conventional accelerators, due  to their intrinsic features, such as high beam charge and short bunch duration. However, the broadband energy spectrum of these proton sources is a bottleneck that precludes their use in applications requiring a more reduced energy spread. Consequently, in recent times strong effort has been put to overcome these limits and to develop laser-driven proton beamlines with low energy spread. In this paper, we report on beam dynamics simulations aiming at optimizing a laser-driven beamline - i.e. a laser-based proton source coupled to conventional magnetic beam manipulation devices - producing protons with a reduced energy spread, usable for applications. The energy range of investigation goes from 2 to 20 MeV, i.e. the typical proton energies that can be routinely obtained using commercial TW-power class laser systems. Our beamline design is capable of reducing the energy spread below 20%, still keeping the overall transmission efficiency around 1% and producing a proton spot-size in the range of 10 mm^2^. We briefly discuss the results in the context of applications in the domain of Cultural Heritage.

## Introduction

Laser-based proton sources, as generated by irradiating a solid target with a short, energetic laser pulse at high intensity $(I > {10}^{18}W\cdot c{m}^{-2})$ is a topic of intense research. Today, many commercial table-top laser systems in the hundreds of TW-power range (even up to PW-power range), provide laser pulses with energies of up to a few tens of J^1,2^, a duration of 25–30 fs and a repetition rate of up to 10 Hz^3–5^, highly suited for this kind of particle acceleration. One of the current challenges in laser-driven proton acceleration is to improve the characteristics of the laser-plasma based proton sources and make them suitable for practical applications, where most efforts are focused on achieving high beam energies and enhanced beam parameters. The features of laser-accelerated protons are complementary, and in some cases even outperforming, to those of conventional accelerators: the energy of proton beams accelerated with high-power lasers has reached the tens of MeV^3,6–9^, the bunch duration at the source is in the range of picoseconds and the bunch charge goes up to $\sim {10}^{13}$ particles per shot^10,11^. Up to now, the most consolidated acceleration mechanism is called Target Normal Sheath Acceleration (TNSA)^12,13^. This regime allows achieving routinely a maximum energy in the range of 10–20 MeV (for the commercial, high-repetition rate, TW-class laser systems) and even up to 85 MeV (the highest energy achieved recently) in the case of PW laser facilities^8,9,14–16^.

With their characteristics, laser-driven proton sources have demonstrated to be highly promising for many innovative applications including ultra-fast radiography^17,18^, isochoric heating (warm dense matter)^14,19,20^, medical applications^21,22^, injectors for conventional accelerators^23^, improved analysis on cultural heritage^24^, material science^25^ or advanced materials synthesis^26^. However, for many applications, such as in the medical field, the maximum ion energy and the maximum ion flux are still too low and the energy spread of the beam is too large, since it reaches 100% in the TNSA acceleration regime. Innovative acceleration mechanisms (as an alternative to TNSA) are being explored in order to improve the beam quality (energy spread and divergence) and acceleration efficiency: e.g. the Shock-Wave acceleration regime^15,27^ or the Break Out Afterburner^28^ mechanism. However, compared to TNSA, these acceleration mechanisms require more stringent conditions and are less consolidated. Furthermore, improvements at the source have been suggested using special target engineering^6,29,30^. The use of special targets, however, represents additional challenges and costs, which make them difficult to implement on a routinely basis and on higher repetition rate laser systems.

Up to recent, most effort in the field was put in improving the acceleration mechanism at the source, i.e. the laser-matter interaction. It is only in the last years that both the laser-plasma and accelerator community are heavily working towards making these laser-generated beams more reliable, stable and easy to operate, going towards developing laser-driven particles beamlines, including proton beamlines. For obtaining laser-accelerated proton beams suitable for applications, the combination with conventional beam manipulation devices represents a possible, easily implementable solution^23,31^, in particular if commercial laser systems and standard targets are used. The so-called hybrid beamlines make use of conventional beam transport, manipulation and focusing elements and allow improving the parameters, the stability and the reliability of the laser-plasma particle source. Some of their typical requirements are: 1) being able to lower the energy spread of the ion beams down to a few percent around the central energy, 2) providing a reproducible, reliable, high repetition rate beam (multi-Hz repetition rate), 3) having compact dimensions, using devices that are easy to implement.

The first experimental evidence of a laser-driven beam line that involves conventional accelerator devices has been provided by Nakamura *et al*., who used a 40 kV/20 mm Radiofrequency (RF) resonator to post-accelerate a proton beam with energies in the multi-hundred keV range, obtaining a final energy spread of about 7%^32^. A more complex beamline, involving the post-acceleration by a RF cavity, has been implemented by Nishiuichi *et al*. in 2010: The central energy of the obtained proton beam was 1.9 MeV, obtained with a laser-plasma source driven by a 630 mJ, 45 fs long laser pulse^33^. The use of accelerating cavities for the post-acceleration of TNSA beams with higher energy has been proposed by Antici *et al*. between 2008 and 2011 by using accelerating cavities that are commonly used in conventional proton accelerator facilities, i.e. drift-tube-linac (DTL) cells. The proton beam that has been considered had an initial energy of 7 MeV and numerical calculations showed how the bunch could be accelerated up to ∼15  MeV with the use of 48 DTL cells, over a distance of 8 m^23,31^.

There have been also studies about the collimation of laser-generated proton beams using conventional focusing devices such as quadrupoles and solenoids. Quadrupoles for the focusing and monochromatization of a laser-accelerated proton beam have been used by Ter-Avetisyan *et al*. and Schollmeier *et al*., in 2008^34,35^. The first group managed to select and focus $\sim {10}^{8}$ protons, with a central energy of 3.7 MeV, out of a TNSA beam generated by a TW-class laser. The latter group used permanent magnet quadrupoles, such as those of ref.^36^, to focus a 14 MeV proton beam accelerated with a PW-power class laser. The implementation of high-power solenoids, downstream a laser-plasma proton source, has been tested by Burris-Mog *et al*.^37^ and Busold *et al*.^38,39^, performing experiments that involved magnetic solenoid fields in the multi-Tesla range, also in combination with RF cavities for phase-space rotation^40^.

Devices that account for the energy selection of TNSA proton beams have been studied and are reported in refs.^41,42^. These energy selectors have been designed for either low-energy ranges (mainly below 1 MeV in ref.^41^) or higher energies (up to 60 MeV in ref.^42^) that are currently at the upper limit of the typical energy spectrum obtained using TNSA. For the latter case, the physical dimensions are large and even if the covered energy range is wide, they seem to be quite out of reach for the typical TW-laser experimental chambers. Furthermore, this kind of energy selection device requires a narrow divergence of the incoming proton beam in order to work efficiently and, therefore, requires to be further optimized by being coupled with a focusing system of the proton beam. In ref.^43^ the implementation of magnetic quadrupoles for injecting the beam into an energy selector is proposed, but the considered energy range (up to 60 MeV) is higher than what can be easily and routinely obtained with commercially available high-repetition rate lasers and the length of the beamline (>5 m) is at the higher-end of the typical dimensions for a standard experimental setup.

In this paper we provide guidelines for the design and optimization of a compact beamline driven by proton beams laser-accelerated at the source by the standard TNSA regime. The design includes both, a beam collimation stage based on beam focusing devices (permanent-magnet quadrupoles (PMQ)) and an energy selection device. We propose optimized beam line lattice structures for different mean proton beam energies in order to maximize the transport of the beam and, as such, the efficiency of the entire beamline. We focus on an energy range of 2–20 MeV, such as currently routinely obtained by commercially available high power multi-hundred TW laser system using different acceleration mechanisms. In our study we aim at maximizing the energy transport and achieving a final proton energy spread of <20%, which would represent a significant improvement for many of the above mentioned applications.

The analysis of the beamline has been performed with a methodology similar to that employed in conventional accelerators, i.e. using codes that are standard for conventional accelerator beam dynamics: TSTEP (a derivative of PARMELA^44^) and TRACE3D^45^ for the particle tracking and SUPERFISH^46^ for the magnetic properties of the beamline elements.

Using a parametric approach, we have tested the effect of the focusing quadrupoles and of an energy selector device on the overall efficiency (performance) of the beamline.

**Figure 1:**
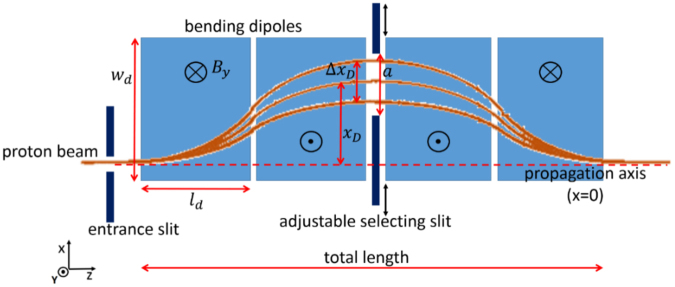
Scheme of the selection device based on conventional magnetic dipoles. The proton beam is bent throughout the magnetic chicane and the particle dispersion reaches its maximum between the second and third dipole. The selecting slit can be adjusted transversely in both position and aperture.

At first, we start the analysis with the energy selection device, since we need to characterize the energy selection process and the acceptance of the device for the incoming protons, in particular in terms of acceptance entry angle (laser-accelerated particles have a large divergence angle which makes them much more difficult to capture). We then concentrate on how the selector parameters (i.e. the length of the dipoles ${l}_{d}$, their width ${w}_{d}$ and the strength of the magnetic field $B$ (see Fig. 1)) and the initial parameters of the incoming beam have an impact on the energy selection process, in terms of final energy bandwidth and transmission efficiency. Once that the relevant beam parameters have been identified, we investigate the required characteristics of the focusing quadrupoles in terms of magnetic field gradient, magnetic length and spacing between them, in order to maximize the overall efficiency of the capture and transport the beam from the source to the energy selection device. We show that an optimized beamline design allows for an overall efficiency of ∼1%, considering a final proton energy spread of <20%. Intermediate solutions are proposed and discussed.

### Parametric analysis and optimization of the energy selection device

The design of our energy selection (ES) device consists of a magnetic chicane built with four permanent magnet dipoles (having length ${l}_{d}$ and width ${w}_{d}$), one entrance slit and one selecting slit, similarly to that reported in refs.^41,42^. A qualitative scheme of such a device is shown in Fig. 1. We define the transmission efficiency of the ES as the ratio between the particles coming out of the ES and the particles that enter the first collimation slit, at the selected central energy of the final beam. Thus, the efficiency is ${\eta }_{ES}={N}_{ES}^{out}/{N}_{ES}^{in}$.

The four dipoles are placed symmetrically with respect to the central selecting slit, allowing the proton beam to return on its original propagation axis (corresponding to the trajectory a particle with infinite energy) at the exit of the selector. The dipoles displace the particles from the propagation axis according to their energy, as the beam rigidity is $B\rho =p/e$ ($B$ is the strength of the magnetic field of the bending dipoles, $\rho$ and $p$ are the bending radius and the momentum of the particles, respectively, and $e$ is the elementary charge). The particles are displaced from the propagation axis by a distance ${x}_{D}$ that depends from their energy. By placing the selecting slit (movable along the x-axis) between the second and third dipole, it is possible to select different central energies of the beam. However, due to the spatial aperture *a* of the slit, particles with a different energy from the reference one are able to cross the slit as well: all the particles of the beam with ${\rm{\Delta }}{x}_{D}=D\cdot \frac{{\rm{\Delta }}p}{p}\le a$ pass through the ES, where ${\rm{\Delta }}p$ is the momentum offset of the particle with respect to the central momentum $p$. $D$ is the dispersion function of the magnetic chicane. The slit at the entrance of the ES is required to reduce the divergence of the incoming beam, which is in the order of tens of degrees at the source^3,14^. This is necessary to avoid that the intrinsic divergence of the particles influences their transverse position along the ES and alters the energy selection process.

**Figure 2:**
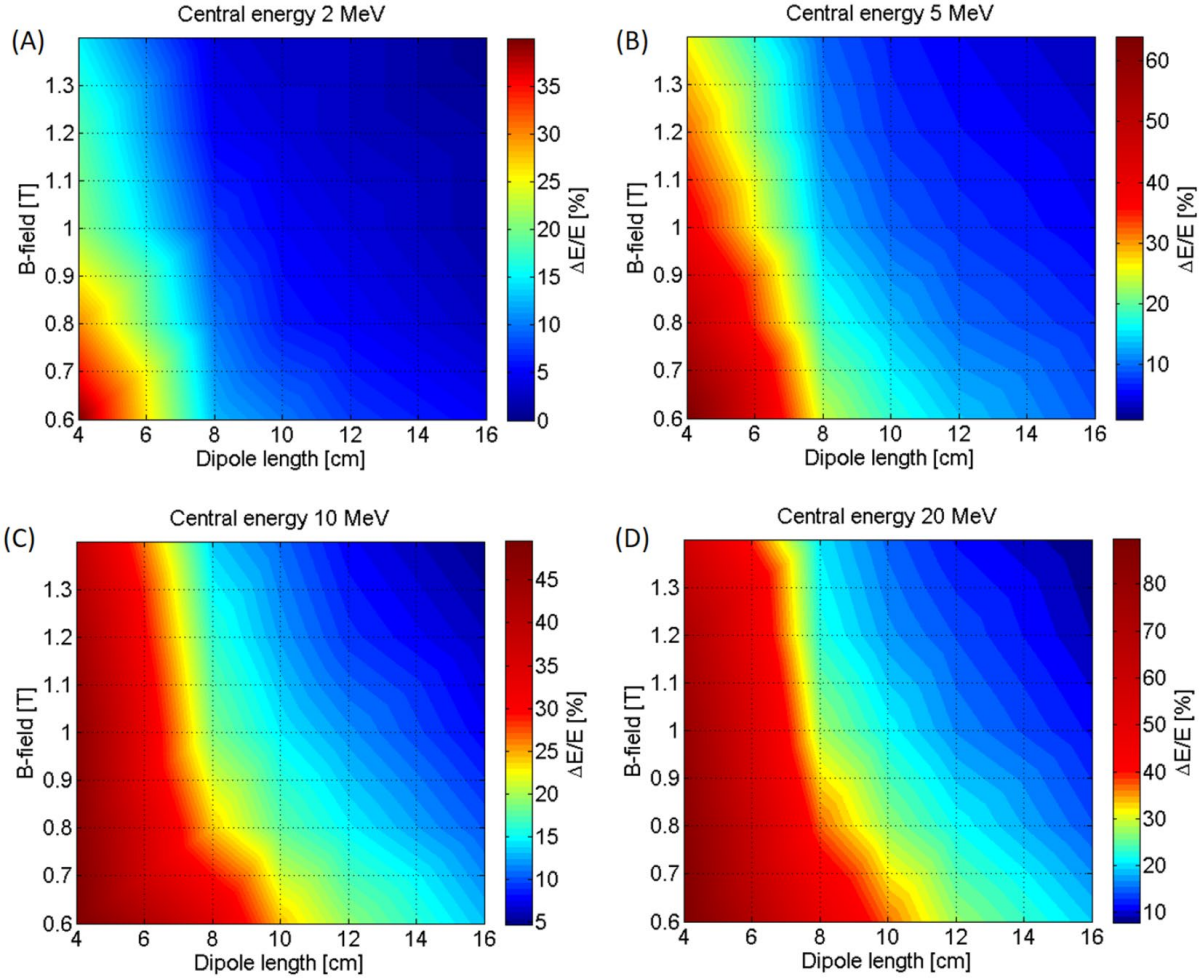
Final energy spread of the proton beam (indicated in the color bar) at the exit of the ES, as a function of the dipole length (x-axis) and magnetic field (y-axis). The maps indicate different mean energies of the selected beam ((**A**) 2 MeV, (**B**) 5 MeV, (**C**) 10 MeV and (**D**) 20 MeV). The initial divergence of the beam is 3 mrad half-angle for all the cases and the aperture of the selecting slit is 500 µm. By fixing the desired final energy spread value it is possible to identify in the maps the required combination of ${l}_{d}$ and B related to the working point.

We have optimized the compactness of the ES by investigating the influence of ${w}_{d}$ and ${l}_{d}$ on the energy selection process. The total length and the width of the device are key aspects in those circumstances where the interaction chamber used for the experiment has a limited available space. The data shown in Fig. 2 have been obtained with the particle tracking code TSTEP. We have simulated the setup of Fig. 1 using an input particle distribution with an initial uniform energy spread ${\rm{\Delta }}E/{E}_{0}$ (where ${E}_{0}$ is the central energy of the beam) of 100%. The proton bunch has a transverse dimension of 500 µm (with a uniform transverse distribution) and a divergence of 3 mrad (half-angle, transverse position-correlated) on the x-plane. The initial spot-size and divergence of the beam are given by the aperture and the distance of the entrance slit from the source (in this case, the 500 µm wide slit is 8 cm away from the source, a comfortable distance for the typical dimensions of a small/medium sized target chamber). The selection slit between the second and third dipole is 500 µm wide. The color-maps of Fig. 2 show the final energy spread of the proton beam (i.e. after passing through the energy selector) as a function of the length of the dipoles and the magnetic field. The plots that are reported address different selected energies (i.e. 2, 5, 10 and 20 MeV) within the range of our interest.

The simulations show that longer dipoles provide the ability to select a narrower energy spectrum. For the case of a 10 MeV central energy (plot 2C)), fixing the magnetic field at 1 T, with four 16 cm long dipoles we obtain a final energy spread of about 5% Full-Width-Half-Maximum (FWHM). Whereas with 10 cm long dipoles the final energy spread is ∼15% (FWHM). Using dipoles with a length shorter than 6 cm is a viable option (provided that the magnetic field is strong enough, i.e. $\ge$1 T) only for selecting central energies below 5 MeV (see the plots of Fig. 2A, B), and if a final energy spread of a few tens of percent is required. The plot 2C, where an energy of 10 MeV is selected, shows that even if using a magnetic field of 1.4 T, 6 cm long dipoles lead to a final energy spread of >25%. The maximum strength of the dipoles’ axial magnetic field ${B}_{y}$ that we have considered is limited by the specifications of commercially available rare-earth materials. As reported by many manufacturers, NdFeB sintered magnets represent the state of the art for permanent-magnet-based devices. Commercial magnets built of this rare-earth material can reach a remanence B-field of up to 1.45 T^47^. We took this value as an upper limit for our simulations and performed simulations with field strengths going from 0.6 to 1.4 T, in order to cover all reasonable values for the magnetic dipoles of the ES (we consider values below 0.6 T as too low for selecting energies as high as 20 MeV, using dipoles with compact dimensions).

The results of Fig. 2 show how the use of dipoles with different axial B-fields influences the energy spectrum of the selected proton beam. A stronger magnetic field induces a greater spatial displacement of the particles along the x-axis (see Fig. 1) even for higher-energy protons, allowing an effective energy selection up to 20 MeV. From Fig. 2D it can be seen that using, for example, 8 cm long dipoles, a ${B}_{y}$ field $\ge$1 T is necessary in order to obtain a final energy spread below 30%. Even if dipoles with a stronger B-field lead to higher manufacturing costs, they represent a solution when a narrow final energy spread is required and when it is not possible to increase the length of the dipoles. In order to produce a beam with a final energy spread of ≤20%, Fig. 2 provides possible combinations for the design of an ES. For keeping the dimensions of the device compact, we have chosen to fix the maximum length of the dipoles at 10 cm and to use a magnetic field of ∼0.95 T. The parameters of Table 1 represent a viable scheme for the ES in the energy range 2–20 MeV.

**Table 1:** Optimized combination of the selector parameters in order to obtain a final energy spread <20% for the energy range 2–20 MeV. The considered initial divergence is 3 mrad half-angle.

Dipole length ${l}_{d}$	10 cm
Dipole width ${w}_{d}$	8 cm
Magnetic field ${B}_{y}$	∼0.95 T
Width of the selecting slit $a$	500 µm

The choice of the width of the selecting slit must be a compromise between the proton flux at the end of the ES and the achieved final energy spread. A wide aperture of the selecting slit is preferable when the flux of protons is a relevant constraint and the requirement concerning the final energy spread is more relaxed. A small aperture, on the contrary, provides a better energy selection of higher energies where the displacement of the particles from the central axis is less. For our case, we have chosen a 500 µm aperture slit as a compromise between effective selection and high transmission efficiency.

**Figure 3:**
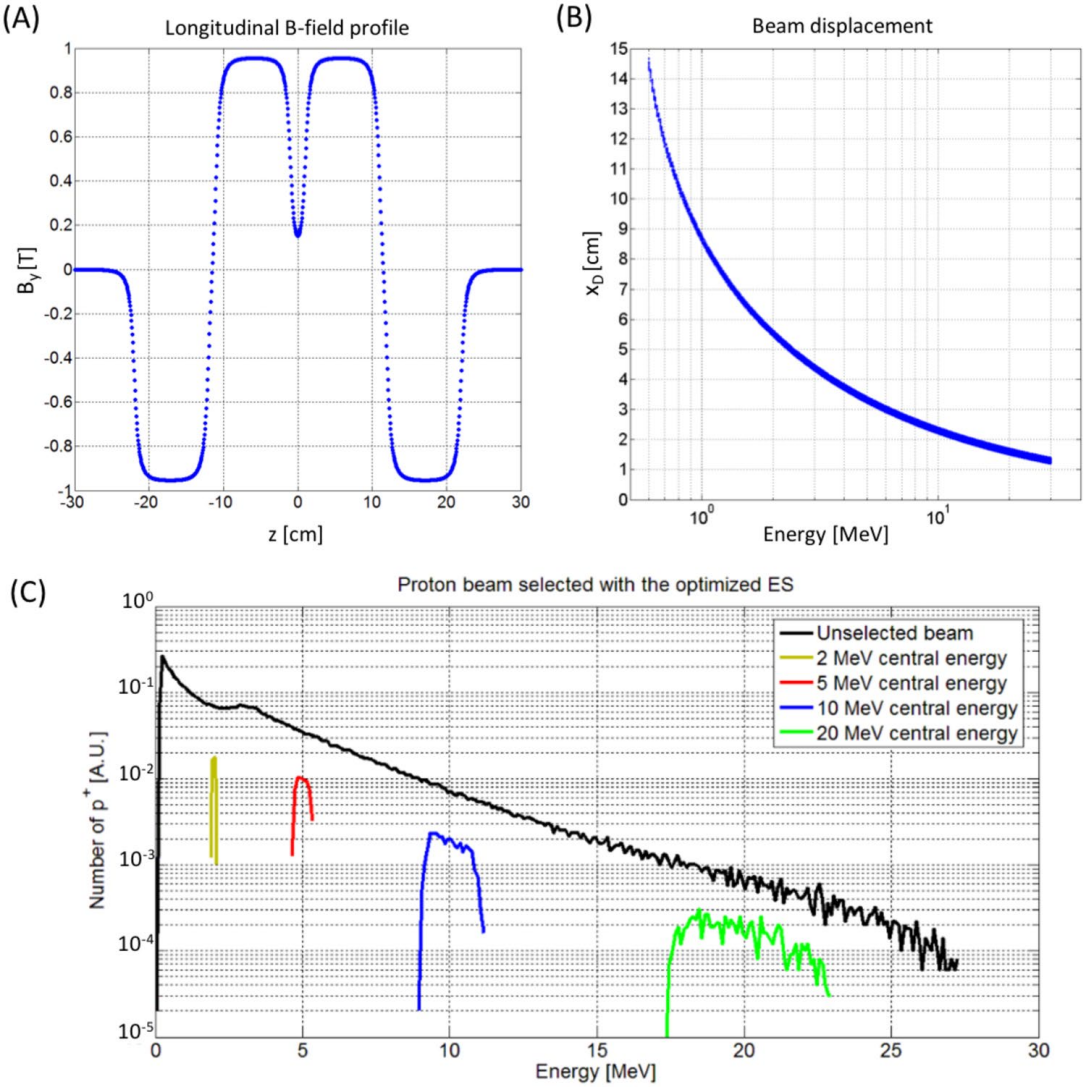
(**A**) ${B}_{y}$-field profile along the four dipoles of the ES, as a function of the longitudinal axis z. The maximum value of ${B}_{y}$ reaches $\sim$0.95 T. The profile, which includes fringe fields at the edges of the dipoles, has been imported into the particle tracking code, in order to run realistic simulations of the ES. (**B**) Displacement of the protons in the x direction, calculated at the longitudinal position where the selecting slit is positioned, i.e. where the beam dispersion is maximum. The displacement is reported as a function of the proton energy. (**C**) Initial (black curve) and final (colored curves) energy spread of the proton beam as obtained with an ES having the parameters reported in Table 1. The selected energies are 2, 5, 10, 20 MeV and the final energy spread is <20% for all the cases. For this set of simulations, a collimated beam with a divergence of 3 mrad has been used, i.e. the losses induced by the collimating slit at the entrance of the ES are not taken into account, leading to an efficiency of the ES of η_ES_ ≈ 20%.

We have performed additional particle tracking simulations by using the set of parameters of Table 1 and by importing in the TSTEP code a realistic magnetic field profile of the chicane, which is reported in Fig. 3A. The set of four dipoles has been simulated with the extension of the SUPERFISH^46^ code called PANDIRA using the parameters of commercially available rare-earth-materials^47^. We have simulated 10 cm long, NeFeB permanent magnets separated by a 1 cm gap. The obtained field profile, used for the particle tracking simulations, is shown in Fig. 3A.

Figure 3B and C show particle tracking simulation results concerning the displacement of the protons as a function of their energy, when the protons travel from the second dipole to the third one (i.e. where the selecting slit is located), and the final energy spectrum of the beam at the exit of the ES. From the data of Fig. 3B it is possible to calibrate the position of the selecting slit for the desired selected energy and to evaluate the lower energy range of the ES: the displacement of the particles must not exceed the width ${w}_{d}$. Using 8 cm wide dipoles, the lower energy range is $\sim$1 MeV (given that the four dipoles are placed all on the same longitudinal axis), i.e. the energy of the particles that are displaced by $\sim$8 cm along the x-axis (see Fig. 1).

Figure 3C shows the final energy spread of the TNSA proton beam before and after passing through the ES (using the field profile of Fig. 3A and the parameters of Table 1). For this set of simulations we have used a realistic input distribution (black curve) having the typical energy spectrum of a TNSA beam. We have used the data of a proton energy spectrum, as obtained from a 2D-PIC simulation^48^, taking into account the typical features of a commercial TW-class laser system: 800 nm wavelength, 30 fs laser-pulse, $\sim$2 J laser energy impinging on a solid target made of aluminum with a thickness of hundreds of nm. The distribution reproduces the exponential decay of the proton number with increasing energy. We have adapted the value of the maximum proton energy to the range of our interest, making it coherent with what has been found in several proton acceleration experiments on TW-class lasers^3,12,14,49^.

**Figure 4:**
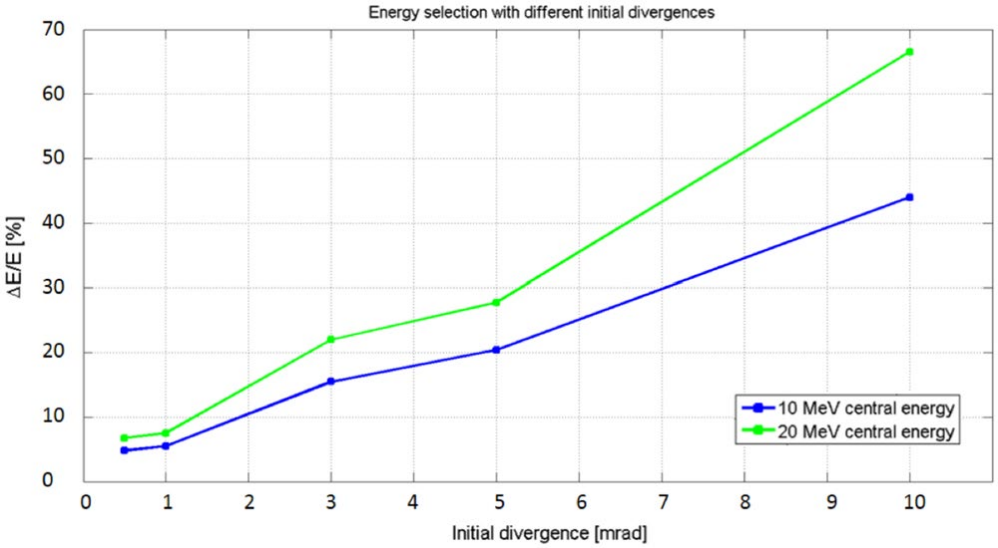
Final energy spread the selected beam as a function of the initial divergence of the protons, for the cases of a mean energy of 10 and 20 MeV. The cases of 0.5, 1, 3, 5 and 10 mrad have been analyzed with TSTEP simulations. The green and blue curve have been obtained by interpolating the values of the final energy spread as obtained by the simulations.

The selection device manages to achieve a final energy spread of ≤20% FWHM for all central energies within the desired energy range of 2–20 MeV. The energy spread after the selection process is 6.25%, 11.5%, 15.25% and 19.89% FWHM for a central energy of 2, 5, 10 and 20 MeV respectively. These results are obtained for an initial divergence of the proton beam of 3 mrad half angle. The efficiency of the device is ${\eta }_{ES}\,\approx$ 20% for all four central energies. This does not represent the efficiency of the entire beam line since the value is calculated by taking as input parameter, i.e. for ${N}_{ES}^{in}$, the number of particles that enter the initial slit that is collimating beam (see Fig. 1), and not the number that is provided by the laser-plasma source. The overall efficiency of the beam line ${\eta }_{BL}$ can be estimated as ${\eta }_{BL}={\eta }_{slit}\cdot {\eta }_{ES}$, where ${\eta }_{ES}$ characterizes the transmission of the ES only (not the entire capture/transport section and the TNSA source) and ${\eta }_{slit}$ accounts for the losses induced by the initial collimation provided by the slit. Using a simple slit for blocking the protons with a divergence larger than the acceptable value (i.e. a few mrad, see Fig. 4) is a possible solution for obtaining a collimated beam, at the cost of losing a part of the protons stemming out of the laser-plasma source. The aperture of the entrance slit should be a compromise between the number of particles that are necessary for a given application and the required final energy spread.

For estimating the losses when collimating the beam with a slit, we can take as reference the intrinsic divergence that the protons have at the laser-plasma source. The TNSA beam typically has a mean divergence of about 15° half-angle^3,14^ (we consider a divergence value averaged over the entire proton spectrum), corresponding to a solid angle of about 0.21 steradiant. The initial slit reduces the horizontal divergence to a value of 3 mrad and the vertical divergence is given by the high of the dipoles’ gap, 1 cm in our case with a distance of 8 cm from the source, giving a 56 mrad half-angle divergence. This collimation leads to a value of ${\eta }_{slit}\approx 0.37 \%$ and an overall efficiency of the beamline of ${\eta }_{BL}\approx 0.074 \%$.

Using the particle tracking code, we have studied the effect of the initial divergence of the incoming proton beam on the energy selection process. At the source, protons are accelerated within a cone with an aperture of tens of degrees with a non-uniform angular distribution (the protons with maximum energy are accelerated within a narrower cone^3,14^). Collimating the beam before the ES entrance improves the energy selection process significantly^41–43^.

Even after collimation, a beam divergence of a few mrad has a relevant effect on the energy selection process. Especially at higher energies (above ∼10  MeV), the intrinsic transverse momentum of the particles induces an additional displacement of the beam trajectory, interfering with the dipoles’ dispersion: increasing the initial divergence from 5 mrad to 10 mrad leads to an increase of the final energy spread of a 10 MeV beam by more than a factor two, as reported in Fig. 4, where we show the behavior of the ES when selecting proton beams with different initial divergences. We show the cases of 10 and 20 MeV, since at lower energies the initial divergence has less influence on the selection process (the displacement induced by the dipoles dominates over the intrinsic divergence of the particles). All the simulations have been run with the following parameters of the ES: 10 cm long dipoles and a field strength of $\sim$1 T. The selecting slit has an aperture of 500 µm for all cases. The interpolated curves of Fig. 4 show the final energy spread of beams with initial divergences that range between 0.5 to 10 mrad (half-angle). These data show that a final energy spread of <20% is achievable only if the divergence is <5 mrad for the 10 MeV case and <3 mrad for the 20 MeV case. The transmission of the ES is improved when the beam is collimated within a tighter angle: for the case of 10 MeV, about 57% of the particles of the incoming beam are transmitted through the ES if the divergence is 0.5 mrad. This value decreases to <10% for the case of a beam with a divergence of 10 mrad. For the other analyzed energies, similar values of efficiency have been obtained.

### Optimization of the beamline using permanent magnet quadrupoles for focusing

As mentioned in the introduction, besides energy selecting the protons, it is advisable to collimate them in order to maximize the efficiency of the beamline. An improved solution for capturing the protons and injecting them into the ES is represented by the use of a focusing system, based on magnetic quadrupoles, placed after the TNSA source that captures the beam and focuses the protons into the entrance slit of the ES.

Using the beam optics code TRACE3D^45^, we have evaluated the optimized parameters of the focusing quadrupoles by running a matching algorithm that gives the optimal values of focusing strength and spacing between the magnetic elements. The aim is to achieve the lowest possible divergence for the protons at the central energy of the beam line. We set goal parameters, in terms of Twiss $\alpha$ and $\beta$, for the focused proton beam at the end of the PMQ array. Optimizing the quadrupoles for *α* = 0 and *β* = 100 m, allows obtaining collimated beam with a divergence of a few mrad at the entrance of the ES. For even lower values of $\beta$, i.e. a tighter focusing of the beam, the algorithm fails to optimize the spacing between the quadrupoles.

**Figure 5:**
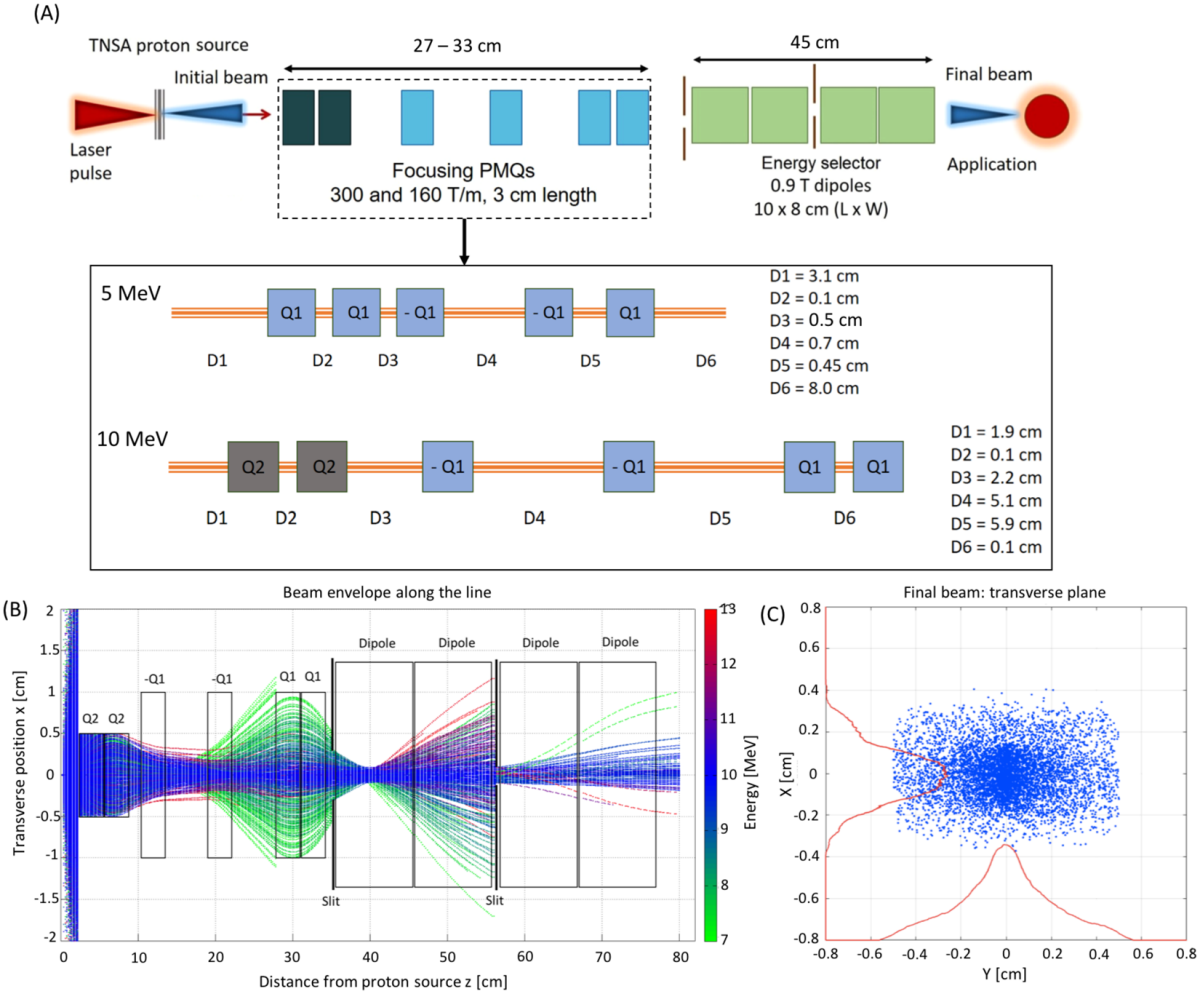
Layout of the complete beamline for the case of a 10 MeV central energy. The design, as reported in (**A**), includes the focusing section made by an array of 6 PMQs. Q1 and Q2 indicate PMQs with a gradient of 160 T/m and 300 T/m, respectively. The dipoles of the ES, represented by the green squares, have the parameters listed in Table 1. The entire beam line, from the laser-plasma source to the exit of the ES, is $\sim$80 cm long. The inset of (**A**) gives further details on the spacing between the PMQs that has been obtained by optimizing the beam line for an energy of 5 and 10 MeV as examples for all. The “−” sign indicates a de-focusing (on the x-axis) quadrupole. The plot (**B**) shows the beam envelope (on the x-z plane) as a function of the distance from the laser-plasma source for the optimized case of 10 MeV, as obtained from a ray-tracing analysis of the TSTEP simulation. We show the particles having an energy between 7 and 13 MeV, identified by the colorbar: the protons around E = 10 MeV are collimated and can be injected into the ES with an improved efficiency. The plot (**C**) shows the transverse shape of the proton beam at the exit of the selector, for the case of 10 MeV mean energy. The transverse distribution of the protons is gaussian-like, in both x and y, as can be seen from the red histograms. The transverse dimensions (FWHM) are $\sim$3 mm in x and $\sim$3.5 mm in y.

At first, we run simulations by keeping both the magnetic field gradient of the quadrupoles and the spacing between them as free parameters. By doing so we were able to evaluate a versatile value of the quadrupoles’ field gradient that allows implementing optimized focusing section for different mean proton energies of the beamline. The data that we have obtained indicate that with two types of quadrupoles, having a field gradient of 160 and 300 T/m (reported in Fig. 5A with Q1 and Q2, respectively), it is possible to capture the particles from the source. These field gradients are required to compensate the high divergence of the proton beam at the source (i.e. fraction of radiant) and are achievable with state-of-the-art PMQs based on rare-earth materials^36,47^. The inner diameter of the quadrupoles is 2 cm and 1 cm, respectively, and the length is 3 cm for both cases. For different mean energies of the beamline in the range 2–20 MeV, it is necessary to adjust the combination and the spacing between the PMQs for obtaining an optimized focusing.

We have optimized the spacing between the quadrupoles for a mean energy of 3, 5, 10 and 20 MeV, by running a second set of simulations with TRACE3D, keeping the field gradient of the quadrupoles as fixed variables. We have analyzed several combinations, i.e. sequences, of quadrupoles by running the matching algorithm of TRACE3D and the code delivered an optimized focusing line for the analyzed energies within the range of interest.

In Fig. 5A we report the layout of the complete beam line, including the focusing and energy selection stage, for the cases of 5 and 10 MeV central energy (see inset), as examples for all the cases. The detailed data of the inset have been obtained with TRACE3D and have been checked with TSTEP for an improved accuracy. The beamline relies on the same two types of PMQs even for different energies. It is sufficient to adjust the sequence of the focusing elements in order to configure the beamline for a different energy. If the distance between two adjacent quadrupoles, which is of 1 mm for some cases, is too short for a real case setup, the combination of quadrupoles can be replaced by a single PMQ having twice the magnetic length, i.e. in our case a quadrupole of a length of 6 cm. Fig. 5B shows the result of a ray-tracing analysis of the TSTEP simulation for the case of 10 MeV mean energy. The reported beam envelope (in the x-z plane, i.e. the plane of dispersion of the ES dipoles) shows a collimated beam at the central energy of the beamline, in this case 10 MeV, that has been obtained from TSTEP simulations based on the PMQ parameters optimized with TRACE3D. Along the ES dipoles, the displacement of the beam from the original propagation axis is not visible due to the fact that the position of the particles is calculated with respect to the trajectory of a reference particle at 10 MeV, which travels on the central axis of the beam, i.e. having transverse coordinates $x=y=0$. Only the protons having an energy between 7 and 13 MeV are displayed in order to make the image more readable (the other particles with lower/higher energies are lost in the transport along the PMQs or are eliminated by the ES). In Fig. 5C we report the final transverse dimensions of the proton beam, as obtained at the end of the beamline, i.e. at the exit of the ES. The transverse distribution has a Gaussian shape with a FWHM of $\sim$3 mm in the $x\,$direction and ∼3.5 mm in the $y\,$direction. The beam is well collimated since the final transverse divergence is in the order of a few mrad. When the protons pass through the first two PMQs of the beam line (with a 1 cm aperture, see Fig. 5B), they fill the entire aperture. The non-linearity of the magnetic gradient near to the pole tips of the quadrupoles induces chromatic aberrations. This effect, however, has only little influence on the final spot of the beam since, as visible from the ray-tracing plot, the protons around the central energy of 10 MeV are immediately focused towards the central axis and are subject to a linear field gradient in the following PMQs. These PMQs have a wider aperture (2 cm) and exhibit a linear gradient of 160 T/m up to ∼1 mm distance from the outer edges (as obtained from SUPERFISH simulations). Only the protons having an energy much lower or much higher than 10 MeV, pass near the edges of the following PMQs and suffer significant aberrations; these particles will be eliminated by the ES downstream. Moreover, the final spot of the protons, as shown in Fig. 5C is mainly given by the aperture of the selecting slit, which eliminates the particles that experience strong chromatic effects. In the plots of Fig. 5B and C, we report the case of a 10 MeV beam, as an example for all energies; the beam parameters that we have obtained for the cases of the other analyzed energies (i.e. 3, 5 and 20 MeV), are similar in terms of envelope along the line and final transverse dimensions.

**Figure 6:**
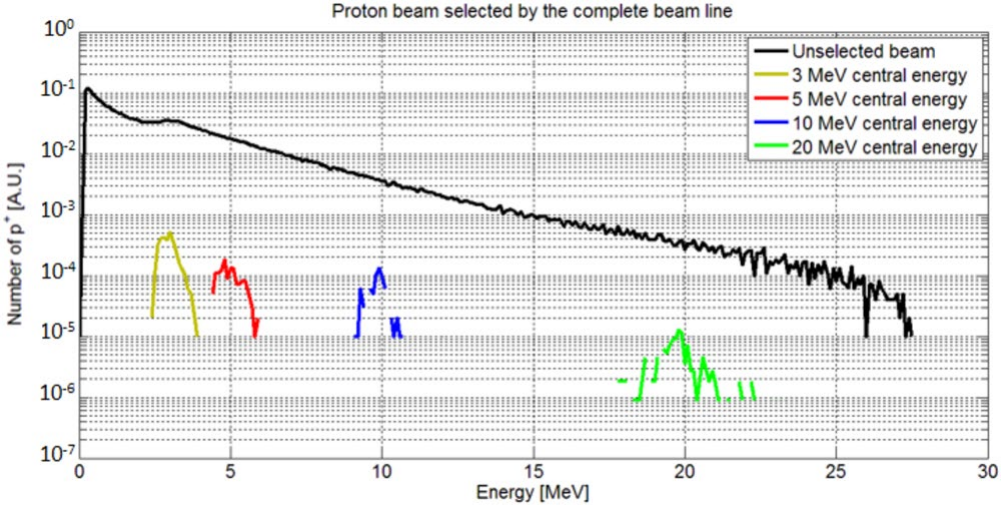
Final energy spread of the proton beam as obtained at the end of the complete beamline, including the focusing PMQ array and the ES. The focusing section has been optimized differently for each central energy. The parameters of the ES, indicated in Table 1, are the same for all energies in the range 2–20 MeV and have been optimized using the data reported in Fig. 2. The obtained efficiency ${\eta }_{BL}\ge 1 \%$ (for all analyzed energies) includes all the losses from the laser-plasma source, i.e. the total TNSA accelerated beam, to the end of the beamline.

For implementing a beamline with a focusing and an energy selection section, we have optimized the devices in the following steps: (I) The parameters of the ES need to be optimized with respect to the required final energy spread. From the data of Fig. 2 we have chosen a set of parameters that allows obtaining a final energy spread of $\le$20%. We have simulated the ES for different energies in the range 2–20 MeV (Fig. 3C). (II) We have evaluated the initial proton beam divergence that is required at the entrance of the ES, in order to obtain the desired final energy spread. The numerical data of Fig. 4 show that in our case an initial divergence of $\le$3 mrad (half-angle) is required. (III) The focusing section of the beam line has been optimized with the aim to collimate the protons to the required divergence. With TRACE3D, we have obtained a different, suitable spacing between the PMQs for each central energy that we have analyzed. (IV) We have simulated the beamline, using TSTEP, coupling the quadrupoles to the ES in order to demonstrate that the focusing section improves the injection of the protons into the initial slit of the ES. The particle tracking simulations with TSTEP allow analyzing the behavior of the complete beamline (see the scheme of Fig. 5A), evaluating all the losses from the laser-plasma proton source to the end of the line. The result of the start-to-end simulations is reported in Fig. 6, where the complete beamline is simulated and the initial and final energy spread of the beam are shown. The initial energy spectrum (indicated with the black line) of the beam has the typical shape of a TNSA proton beam (the energy spectrum we have used as input is the same as the one for the study of the ES only, reported previously in Fig. 3C) and has an energy-dependent divergence distribution, such as the one reported in ref.^14^. The different colored final energy spectra represent the proton beam at the exit of the ES. The quadrupoles in the simulations of Fig. 6 have been optimized, in terms of focusing gradient and spacing, with the matching algorithm of TRACE3D for the central energies 3, 5, 10 and 20 MeV.

From Fig. 6 it can be seen that losses, in terms of final particle number, cannot be avoided but the focusing stage reduces the divergence of the beam and enhances the overall efficiency of the beamline, compared to the case where the laser-generated protons are selected by the entrance slit only. It is important to stress the fact that the efficiency shown in Fig. 3C, differently from this case, only takes into account the losses of the selecting slit in ES (reducing the divergence of the beam using the entrance slit leads to a loss factor of ∼10^4^). The energy spectra of Fig. 6 show how the combination between quadrupoles and ES allows to achieve a final energy spread of ≤20% for all energies. Moreover, the focusing stage allows improving the start-to-end efficiency of the full beam-line to ${\eta }_{BL}={N}_{BL}^{out}/{N}_{source}\ge 1{\rm{ \% }}$ in the entire energy range. In this case we compute all the losses of the beamline, by taking into account the number of protons available (within the selected energy range) at the source and at the exit of the beamline.

### Potential application in the field of Cultural Heritage analysis

Our study of a compact beamline allows investigating the use of these laser-driven protons for applications that require low energy, quasi-monoenergetic beams. The proposed design, which delivers a final beam with an energy spread of <20% (FWHM) and an overall transmission efficiency of about 1% for all the investigated energies (2–20 MeV), is well suited for implementing a compact laser-driven beamline devoted to the analysis of Cultural Heritage (CH) artifacts via Proton Induced X-ray Emission (PIXE). Materials of interest for CH, such as bronze, silver, gold, marble, ceramics, pigments etc, can be irradiated with energetic proton beams (typically with energies of 1–5 MeV) in order to stimulate the emission of X-rays. Information about the chemical composition of the irradiated material can be retrieved from the spectral analysis of the emitted radiation. This consolidated technique is routinely performed using conventional accelerators^50–52^. These facilities, however, produce typically a low-flux proton beam and allow analyzing small spot areas (in the tens of µm^2^ range), requiring a long time for scanning a larger surface (up to thousands of seconds for an area in the order of mm^2^) and therefore potentially causing a damage to the artifact. The basic setup for PIXE analysis at the AGLAE facility located at the Louvre (France), implements an electrostatic accelerator that irradiates a spot area of 10 µm diameter with a charge of 1.8 nC, compiling the analysis over a time of 250 ms. An area of 1 mm^2^ can be scanned with a pencil-scan procedure, requiring 2100 s of time^52^.

The use of laser-accelerated protons as an alternative to the conventional accelerators has been successfully tested in the proof-of-principle experiment of ref.^24^ where the CH samples have been irradiated with a non-manipulated, laser-accelerated proton beam. If coupled to a high-repetition rate laser, the compact, optimized beamline presented in this article potentially represents an improved solution for laser-driven PIXE, producing a high proton flux, a high shot-to-shot stability and a reduced energy spread for an enhanced depth precision.

Novel Ti:Saphire laser systems operate at multi-Hz repetition rate and generate proton bunches in the nC range, as reported in ref.^53^, where the spectrum of the accelerated beam shows $\sim {10}^{11}$ protons/MeV for a mean energy of 3 MeV. This kind of beam can be injected into the optimized beamline allowing to obtain a final mean energy of 3 MeV ($\pm$30  keV energy spread FWHM, which leads to a penetration depth of about 36 $\pm$ 6 µm into a Silver sample, as an example of a material typical of CH artifacts) and, with an overall transmission efficiency of ${\eta }_{BL}$ = 1.2%, a final beam charge of $\sim 7.2\times {10}^{8}$ protons/bunch, i.e. $\sim$ 0.12 nC/bunch. Operating the beamline at a repetition rate of 10 Hz allows irradiating the sample with a proton flux of 1.2 nC/s, scanning an area of about 10 mm^2^ (see Fig. 5C in the previous paragraph, where the beam spot size for an energy of 10 MeV is reported, which, however, is similar to what we have obtained for the other energies of the beam line, including 3 MeV). Since our beam line is capable of delivering over a millimetric area the amount of charge that is necessary to for the PIXE analysis, it is not required to perform a pencil scan for obtaining information about the “global” chemical composition, suggesting that the PIXE analysis of a large surface can be compiled within a few seconds, i.e. a few tens of laser shots on a 10 Hz laser.

These promising results open the way to compact laser-driven beamlines dedicated to the PIXE analysis as an alternative to the large conventional accelerator facilities that are used today.

## Methods

The particle tracking simulations that we have run in order to study the beamline and its components (i.e. the magnetic dipoles, the slits, the PMQs) are performed with the code TSTEP. These simulations have been run with an initial bunch distribution having 100000 marco-particles for the analysis of the ES (i.e. the simulations of Fig. 2) and ∼250000 macro-particles for the analysis of the complete beamline, i.e. both the ES and the focusing section (∼1000000 for the case of 20 MeV central energy, in order to compensate the exponential decrease of the energy spectrum). The ray-tracing analysis of Fig. 5B, which has the scope of qualitatively showing the beam envelope along the beam-line, has been performed using less macro-particles (about 1/10 of the quantities mentioned above) in order to reduce the computational effort. The code computes the 6-dimensional energy-position vector $(x,x^{\prime} ,y,y^{\prime} ,z,E)$ for each particle, along longitudinal spatial steps $\Delta z$. For our simulations we have set the step increase $\Delta z\approx 0.3$ mm. The energy distribution of the simulated beam is represented the black curve of Figs. 5 and 6. The initial divergence of the particles used for the start-to-end simulations, i.e. of Fig. 6, is energy-dependent, as typical for TNSA beams: we have used a curve similar to what is reported in ref.^14^. Space charge effects have not been included in the simulations. These effects (which lead to significantly longer computation times) are negligible before the focusing elements since the proton beam has a large intrinsic initial divergence of tens of degrees and a wide energy spread, i.e. the beam is emittance-dominated and not space-charge dominated. For the focused beam, i.e. the beam after the PMQ array, up to 90% of the particles are lost and, therefore, the beam current is strongly decreased, leading to very weak space charge effects. The elements of the beamline have been simulated using ideal models (e.g. ideal dipoles not including fringe fields) that are included in the particle tracking code database^54^, only for the study of the ES optimization (see Fig. 2). For all the other simulation sets, we have imported the magnetic field profiles of the beamline devices from the results of more realistic electromagnetic simulations.

The electromagnetic simulations of the beamline elements have been performed with the code SUPERFISH, which is a standard code for analyzing components of conventional accelerator beamlines. This code is capable of solving magnetostatic problems (as it is the case for the permanent magnet dipoles and quadrupoles of the beamline) in a bi-dimensional domain, allowing to obtain realistic field profiles if the parameters of the magnetic material are provided. For the selector dipoles we have simulated NeFeB sintered permanent magnets as they are available commercially, i.e. with a magnetic remanence of ∼11.5 kG and a coercivity of ∼17  kOe. The values used for these cases can be easily obtained from commercial permanent magnets data sheets, such as from ref.^47^. The thickness of the magnetic plates is of 2 cm, they are separated by a 1 cm gap and are surrounded by a 3 cm thick iron yoke.

The beamline’s PMQs are based on a design of the Halbach type^46^ with 12 sectors. The bore radius is 1 cm for the 160 T/m quadrupole (Q1) and 0.5 cm for the 300 T/m quadrupole (Q2). The outer radius is of 4 cm for both cases. For the NeFeB magnetic sectors of the Q1 PMQ we have used a magnetic remanence of ∼11  kG (∼10  kG for Q2) and a coercivity of ∼15 kOe (∼14  kOe for Q2). For obtaining a sufficiently high accuracy of our simulations, we have used a size of the 2D mesh of $dx$ = $dy$ = 0.5 mm for all the simulations.

Before performing the realistic and more accurate particle tracking and/or electromagnetic simulations, the spacing and the optimized parameters of the beamline devices have been preliminarily evaluated using TRACE3D, which accounts for the computation of the proton beam envelope equation along the line and provides beam matching routines (see the previous paragraph).

All the post-processing of the simulations data has been performed using Matlab routines/scripts.
